# Determining Toxic Potencies of Water-Soluble Contaminants in Wastewater Influents and Effluent Using Gene Expression Profiling in *C. elegans* as a Bioanalytical Tool

**DOI:** 10.1007/s00244-022-00959-y

**Published:** 2022-10-03

**Authors:** Antoine Karengera, Ilse Verburg, Mark G. Sterken, Joost A. G. Riksen, Albertinka J. Murk, Inez J. T. Dinkla

**Affiliations:** 1 Department of Animal Sciences, Marine Animal Ecology Group, Wageningen University, Droevendaalsesteeg 1, 6708 PB Wageningen, The Netherlands; 2 Wetsus, European Centre of Excellence for Sustainable Water Technology, Oostergoweg 9, 8911 MA Leeuwarden, The Netherlands; 3 Department of Medical Microbiology and Infection Prevention, University Medical Center Groningen, 9713 GZ Groningen, The Netherlands; 4 Plant Sciences, Laboratory of Nematology, Wageningen University, Droevendaalsesteeg 1, 6708 PB Wageningen, The Netherlands

## Abstract

With chemical analysis, it is impossible to qualify and quantify the toxic potency of especially hydrophilic bioactive contaminants. In this study, we applied the nematode *C. elegans* as a model organism for detecting the toxic potency of whole influent wastewater samples. Gene expression in the nematode was used as bioanalytical tool to reveal the presence, type and potency of molecular pathways induced by 24-h exposure to wastewater from a hospital (H), nursing home (N), community (C), and influent (I) and treated effluent (E) from a local wastewater treatment plant. Exposure to influent water significantly altered expression of 464 genes, while only two genes were differentially expressed in nematodes treated with effluent. This indicates a significant decrease in bioactive pollutant-load after wastewater treatment. Surface water receiving the effluent did not induce any genes in exposed nematodes. A subset of 209 genes was differentially expressed in all untreated wastewaters, including cytochromes P450 and C-type lectins related to the nematode’s xenobiotic metabolism and immune response, respectively. Different subsets of genes responded to particular waste streams making them candidates to fingerprint-specific wastewater sources. This study shows that gene expression profiling in *C. elegans* can be used for mechanism-based identification of hydrophilic bioactive compounds and fingerprinting of specific wastewaters. More comprehensive than with chemical analysis, it can demonstrate the effective overall removal of bioactive compounds through wastewater treatment. This bioanalytical tool can also be applied in the process of identification of the bioactive compounds via a process of toxicity identification evaluation.

**Graphical abstract:**

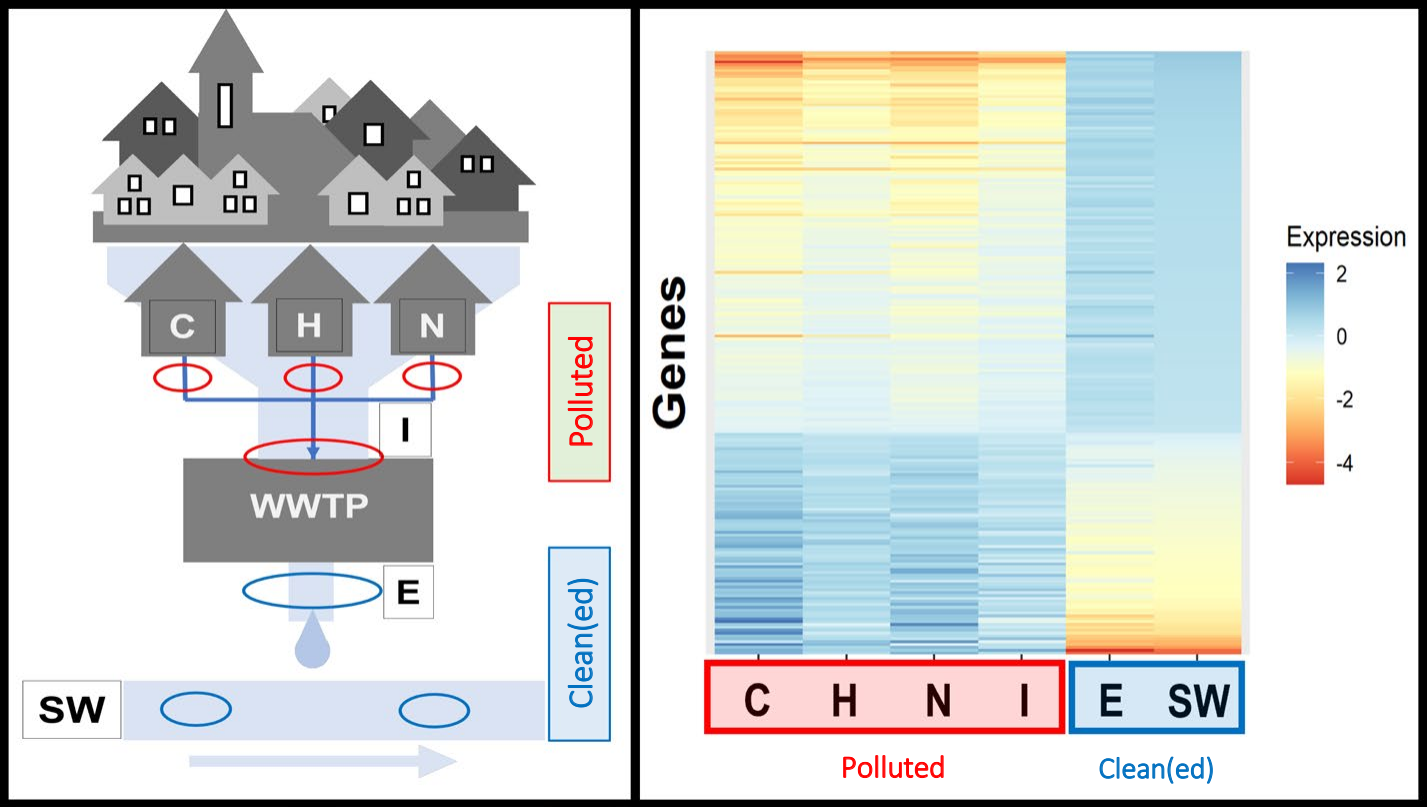

**Supplementary Information:**

The online version contains supplementary material available at 10.1007/s00244-022-00959-y.

A multitude of chemical substances used for anthropogenic activities often end up in municipal wastewater (König et al. 2017; Venkatesan and Halden 2014). Both raw and treated effluents may contain a wide range of natural and synthetic chemicals (Cicek et al. 2007). These substances are usually present as complex mixtures whose composition is difficult to analyze by current chemical methods, among others, because they occur at levels below the limit of detection or no standards are available yet (Schwarzenbach et al. 2006). Substances like hydrophilic compounds are even more challenging for chemical analysis as they are hard to extract or concentrate (Loos et al. 2013). Most of these pollutants, including their metabolites and reaction products, remain unknown and yet they may add to the total toxicological risk posed by the mixture (Stuart et al. 2012).

Municipal wastewaters in the Netherlands are treated in WWTPs, which are generally designed to remove a range of contaminants like suspended solids, phosphorus, nitrogen, biodegradable organic matter, and others (van Beelen 2007). Unfortunately, conventional WWTPs do not completely remove all micropollutants in wastewater (Loos et al. 2013), and many chemicals originating from treated effluents can be found in receiving water bodies like groundwater or surface waters (Margot et al. 2015; Rogowska et al. 2020). Unfortunately, the available analytical methods cannot provide information about the potential toxic effects of these compounds and mixtures thereof (Naidu et al. 2016). Therefore, concerns remain, especially for hydrophilic compounds that may pose environmental health risks or contaminate drinking water sources (Spahr et al. 2020).

Bioanalytical tools, also referred to as bioassays, can quantify the toxic potency of bioactive pollutants in water samples based on their combined effects (Escher et al. 2021; Neale et al. 2020). Bioassays can be in vitro, monitoring responses of cells in culture (Escher et al. 2014) or in vivo, utilizing a whole living system (Wernersson et al. 2015). Most of the existing in vitro and in vivo bioassays are either very specific to one or few biological responses (e.g., endocrine-disrupting activity, aryl hydrocarbon receptor activity, oxidative stress response, and others) or are non-specific indicators of general toxic effects (e.g., mortality, fertility, reproduction, and others) (Escher et al. 2021; Wernersson et al. 2015). Hence, a battery of bioassays is often required for testing various types of bioactive pollutants present in water samples as demonstrated by Jia et al. (2015).

The small nematode *Caenorhabditis elegans* has attracted attention as a model in toxicity testing. This nematode has shown its potential use as toxicological tool for water quality monitoring as shown by Clavijo et al. (2016), where toxicity from pollution in rivers was assessed by measuring effects on *C. elegans* growth. Strengths and limitations for *C. elegans* used in predictive toxicology have been reviewed by Hunt (2017), where good *C. elegans* culture practice (GCeCP) was proposed for reliable and reproducible data. Karengera et al. (2021) recently developed a gene expression-based toxicity bioassay using *C. elegans* as a test organism and showed that the nematodes transcriptomic response can be used to detect the toxic potency of xenobiotics. Toxicity testing by gene expression profiling can provide insights in the type of bioactivity mechanism that is influenced and can be translated toward the nature of the risk the substances present (Fang et al. 2020; Nuwaysir et al. 1999). Also, tests with single contaminants demonstrated that the magnitude of differential gene expression change that were observed can be related to the toxic potency (concentration) that the nematode is exposed to.

In the present study, we aim to evaluate the applicability of the *C. elegans* bioassay for qualification and quantification of the toxic potency of bioactive contaminants present in WWTP influents and effluent. More specifically, the differential gene expression as biomarker for the toxic potency posed by contaminants in wastewater from specific sources was investigated. The samples analyzed in this study were: wastewater from hospital, nursing home, community, and WWTP influent and effluent. In addition, surface water receiving treated effluent was analyzed. Prior to use in nematode exposure, all (waste)water samples were centrifuged and filtered to remove suspended solids. This implies that mainly water-soluble pollutants were present in samples after filtration with only a limited contribution from moderately hydrophobic compounds.

## Material and Methods

### Wastewater Sampling

Wastewater samples were obtained from the sampling campaign as described by Verburg et al. (2019). Briefly, samples were collected from the city of Sneek, in the Netherlands. Wastewater samples from a community of 80 households (C), hospital (H, 300 beds), and nursing home (N, 220 beds) were taken from the receiving wells of which neither received other wastewaters nor rainwater. Wastewater samples originating from these locations were included in our study as they were expected to be severely contaminated with a wide range of pollutants that could present environmental or human health risk. For instance, pharmaceuticals were more likely to be dominant among the chemicals present in the wastewater originating from the hospital facility and from the nursing home to a smaller extent. Irrespective of its source, all wastewaters tested in our study were expected to be polluted with home and personal care products, over-the-counter (OTC) medicines, drugs, pesticides and many others. The sampled wastewater streams (i.e., C, H, and N) each contributed less than 1% of the water inflow into a local municipal WWTP. The main WWTP influent (> 97%) originated from other sources including industrial water, households, stormwater runoff, and seepage from ground and surface waters. The WWTP influent (I) and effluent (E) samples were collected from this WWTP. The WWTP effluent is discharged into an adjacent canal, from which surface water samples were collected upstream (SW1) and downstream (SW2) of the effluent discharge point. In addition, a surface water sample (SW3) was collected from a non-receiving surface water located in a nature reserve, hardly affected by anthropogenic activities. Each sample of 2 L was taken in high-density polyethylene (HDPE) bottles (VWR, Amsterdam, The Netherlands) using an autosampler (except surface waters where grab samples were taken 1 m from the shore at ~ 0.2 m of depth). Time-proportional sampling (24-h samples) was used for C, H, N, I, and E. All samples were transported in cooling boxes and subsequently stored at − 20 °C until use.

### Exposure Media

Prior to the use for exposure, the suspended solid material was removed from water samples by centrifugation and filtration. Therefore, the water-soluble pollutants were the major composition of contaminants left in samples after filtration, whereas the hydrophobic fraction is expected to be very low. Each sample was aliquoted by transferring 10 mL to Falcon™ 15-mL conical centrifuge tubes followed by centrifugation at 3750 rpm for 20 min (Avanti J-15 Centrifuge, Beckman Coulter). Next, the supernatants were further filtrated using Syringe filters Millex^®^ Hydrophilic PTFE (0.45 µm pore size). For all filtrates, pH values in a range of 8.5–9.8 were measured prior to the use for the nematodes exposure. *C. elegans* has been shown previously to be tolerant to such test conditions (Khanna et al. 1997); thus, no pH adjustment was made.

### Nematode Culture and Exposure

Synchronized L4 stage larvae of *C. elegans* wild-type Bristol N2 strain were cultured and exposed in three biological replicates for 24 h as described by Karengera et al. (2022). Prior to commencing with the microarray experiments, we first confirmed visually through a stereomicroscope that the nematodes were alive after the exposure period. For each water sample, approximately 10,000 nematodes were used without feeding during the exposure period. After exposure, the nematode exposure tubes were centrifuged for 1 min at 1000 rpm, 20 °C using a centrifuge (Avanti J-15 Centrifuge, Beckman Coulter). Subsequently, the nematode pellets were transferred into 2-mL microtubes (Eppendorf^®^ Safe-Lock tubes, Biopur^®^) and flash-frozen in liquid nitrogen for 1 min before storing them at − 80 °C until the extraction of RNA.

### RNA Extraction

TRIzol^®^ Reagent with the PureLink^®^ RNA Mini Kit was used to extract total RNA as described by Karengera et al. (2022). Briefly, TRIzol^®^ Reagent was used to prepare nematode lysates from which crude RNA extracts were obtained using chloroform (Molecular Biology Reagent, Thermo Fisher GmbH). The RNA was subsequently isolated from the crude extracts following the manufacturer’s protocol (Thermo Fisher MAN0000406) including column-based RNA isolation through binding, washing, and elution steps. A NanoDrop spectrophotometer was used to measure RNA quantity and quality (Table S1), with an A260/A280 ratio of 1.8 to 2.0 as requirement for further use.

### Microarray Experiments

Microarray analysis was conducted as described before by Karengera et al. (2021) including array preparation, hybridization, scanning, raw data normalization, and pre-processing. Differential gene expression linked to the treatment was investigated by using a linear model, fitted per exposure (i.e., C, N, H, I, and E). The data obtained from SW1, SW2, and SW3 were not significantly different and were therefore used as control. The raw data of this experiment are provided via ArrayExpress (E-MTAB-11260). To identify biological pathways and gene ontologies of differentially expressed genes (DEGs), we analyzed KEGG pathways, gene ontology (GO), and functional domains by using DAVID software v6.8 (Huang et al. 2009). A threshold false discovery rate (FDR) ≤ 0.05 was considered as significantly enriched in the annotation categories.

### RT-qPCR Assays

Gene expression of fifteen target genes selected from microarray data was tested by using RT-qPCR. The cDNA was synthesized from RNA templates via reverse transcription (RT) by using SuperScript™ IV VILO™ Master Mix with ezDNase™ Enzyme as described by Karengera et al. (2022). Two biological replicates were run using the same extracted RNA as used in the microarrays. Due to insufficient RNA material, the third biological replicate sample was run on microarray only and not confirmed by RT-qPCR. PCR primer design and PCR analysis were performed as described by Karengera et al. (2021). Primer sequences used for RT-PCR analysis are provided as supplementary information (Table S2). Raw data were analyzed in Bio-Rad CFX Manager™ Software v3.0, and normalized to *C. elegans* tubulin gamma chain (*tbg-1*) and 14-3-3-like protein (*par-5*) as housekeeping genes.

### Data Analysis and Statistics

Microarray data were statistically analyzed as described by Karengera et al. (2021). Briefly, linear model analysis was used to assess differentially expressed genes (DEGs) per exposure condition whereby a threshold of *p* value < 0.0001 was considered as statistically significant. Custom written scripts for the microarray analysis are provided at https://git.wur.nl/published_papers/karengera_2021_wastewater_fingerprinting. To analyze the variation in gene expression, principal component analysis (PCA) was applied on the log_2_ ratio with the mean expression values using the *prcomp* function in “R” (version 3.5.3, × 64) in RStudio (version 1.1.463).

## Results

### Transcriptome Response to Wastewaters and Treated Effluent

**Fig. 1:**
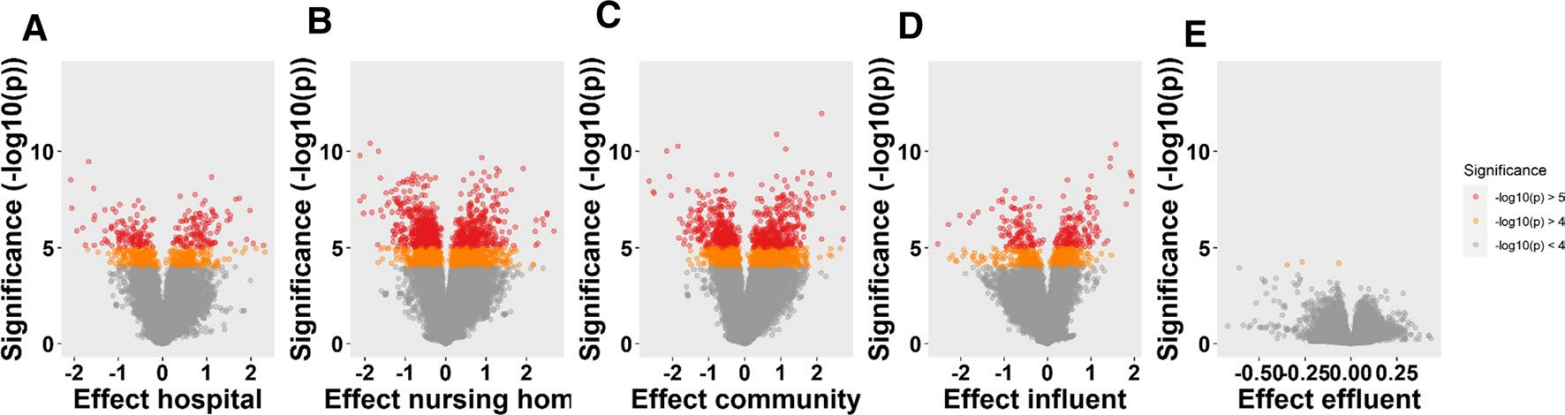
Volcano plots showing the distribution of gene expression changes and p-values. Each dot represents a spot on the microarray, as analyzed by three linear models. On the x-axis the effect is given (a negative sign indicates lower expression over increasing concentrations, a positive sign higher expression over increasing concentrations), on the y-axis the − log_10_(*p* value) obtained from the linear model. These effect plots show an obvious distinction between wastewater samples before and after treatment in a WWTP. Colors provide a visual guide for the thresholds of − log_10_(*p*) > 4 and − log_10_(*p*) > 5. **A** Hospital samples, **B** nursing home samples, **C** community samples, **D** WWTP influent samples, **E** WWTP effluent samples

**Fig. 2:**
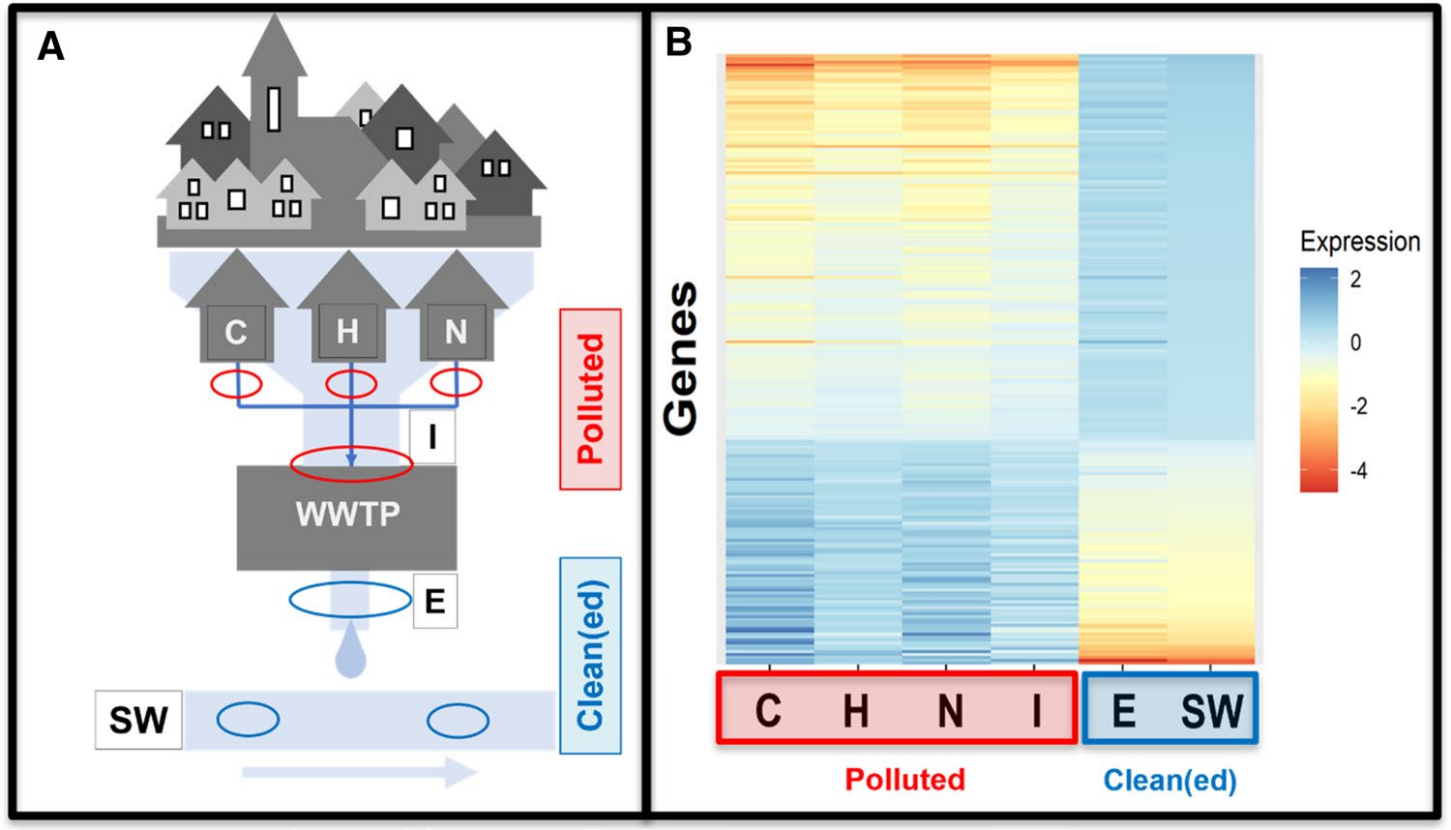
Comparison of gene expression profiles in nematodes treated with (waste)water samples. Sampling points are shown in **A**, including wastewater Community (C), Hospital (H), Nursing home wastewater (N), WWTP influent (I), WWTP effluent (E) and surface water (SW) receiving the treated effluent. **B** Is a heatmap showing the up- (red–orange) and down-regulation (blue) of *C. elegans* genes after exposure to different (waste)water samples. There is a clear difference between gene expression patterns before and after wastewater treatment

**Fig. 3:**
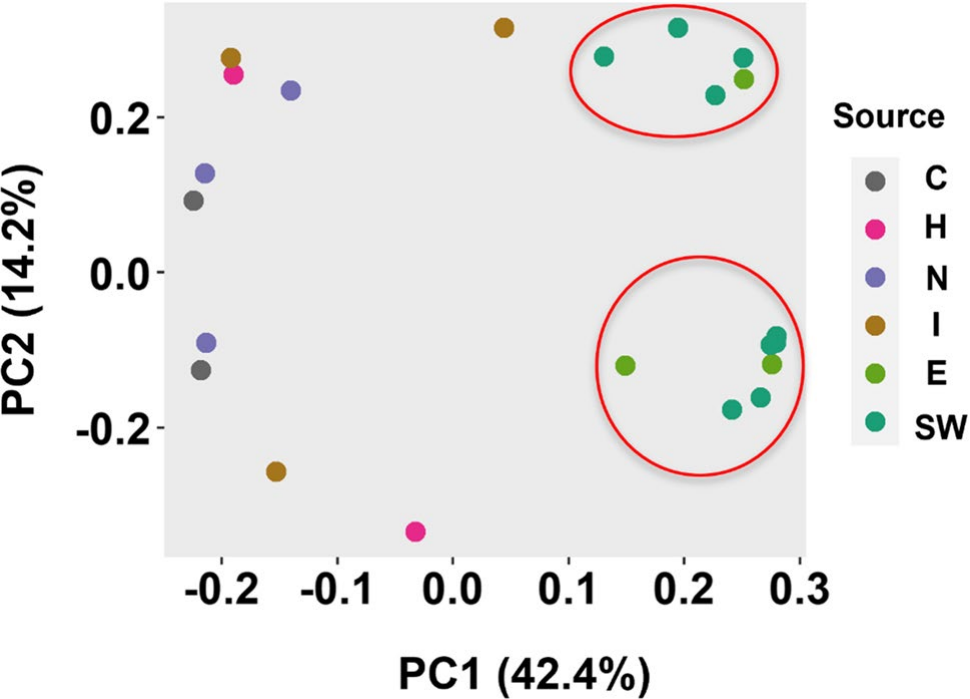
Principal component analysis (PCA) for variation in gene expression. The first two principal components PC1 and PC2 combined captured 56.6% of the variance and mainly separate the surface water and effluent samples from the other samples

**Fig. 4:**
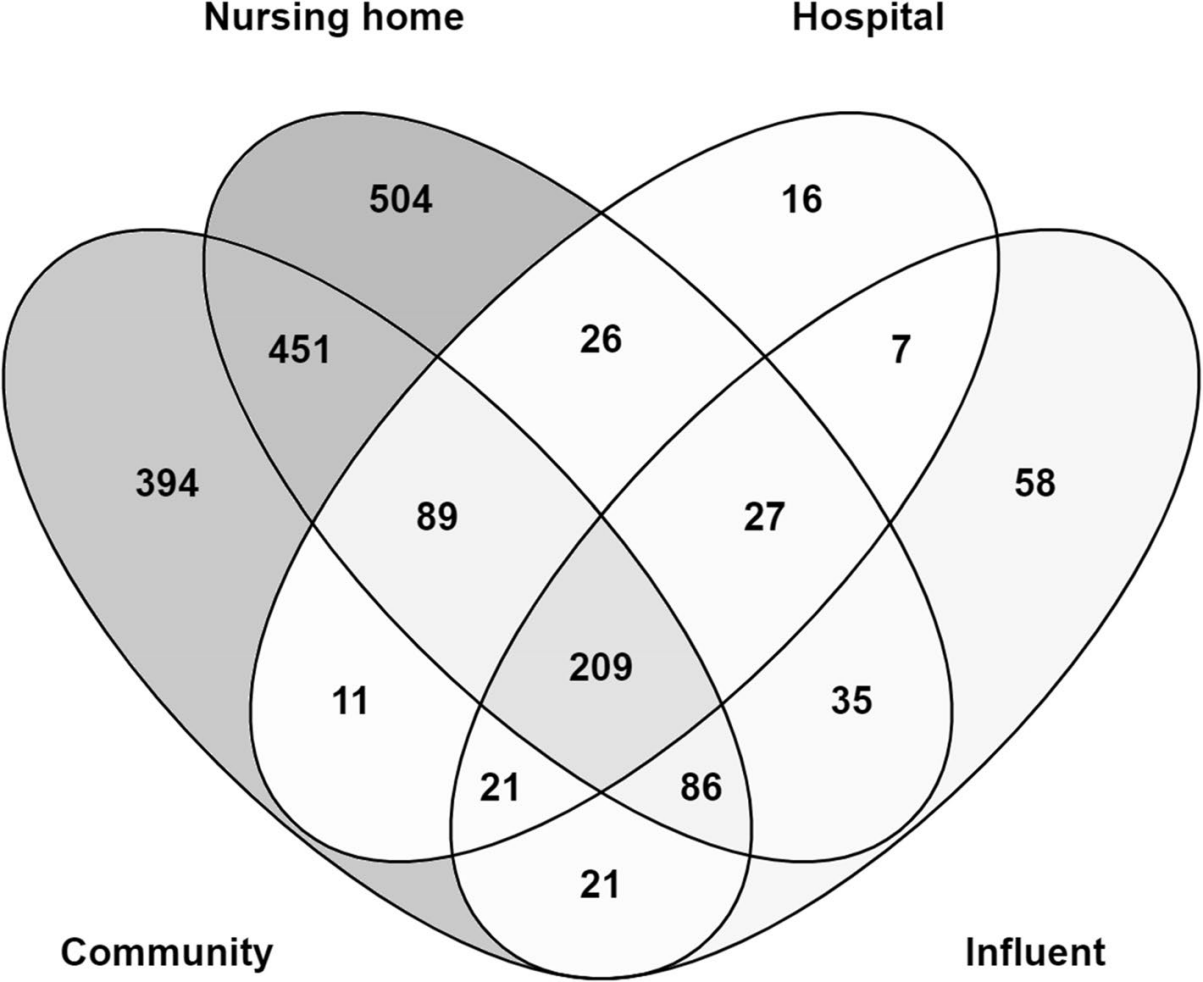
Differences and similarities of genes expression profiles in nematodes after exposure to different wastewater samples. The Venn diagram shows that from the 1756 DEGs (up- or downregulated) in one or more of the polluted samples (i.e., hospital, nursing home, community, and influent), the majority (69%) of these genes were specific to community and/or nursing home wastewaters. The overlap of 209 DEGs (approx. 11%) were found in all polluted samples

The exposed and unexposed nematodes did not show lethality for all tested water samples, as confirmed by visual observation through a stereomicroscope. Whole-transcriptome analysis using microarrays revealed a clear difference between the gene expression patterns induced by wastewater samples before and after wastewater treatment (Fig. 1). Based on the differences in expression profiles, two clusters can be distinguished, one comprising of surface water and E samples and another one comprising of untreated wastewater samples C, N, H, and I (Fig. 2). The difference between the untreated wastewaters and treated effluent or surface water became also clear in principal component analysis (PCA) (Fig. 3). All four wastewater types shared 209 genes that were differentially expressed (Fig. 4), representing 16%, 15%, 51%, and 45% of the total DEGs affected by samples C, N, H, and I, respectively. These genes included those encoding C-type lectin (CLEC) proteins, cytochrome P450 (CYP), and other enzymes involved in xenobiotic biotransformation. In addition, several other overlaps were found between wastewater samples (Fig. 4). C23G10.11 and B0222.4 (known as *spl-2*) genes were found to be the most upregulated transcripts for all wastewater samples. Expression of sphingosine phosphate lyase encoded by *spl-2* is involved in defense responses to gram-positive bacterium. The function of protein encoded by C23G10.11 is not yet known.

**Fig. 5:**
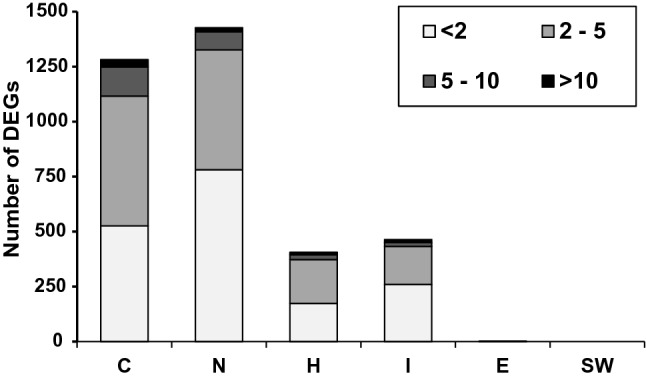
Expression fold change range of differentially expressed genes (DEGs) in the nematodes treated with wastewater samples. Bar charts display the number of DEGs in each fold-change range (i.e., < twofold, twofold–fivefold, fivefold–tenfold, and > tenfold) of the transcription levels induced in the nematodes treated with the samples originating from community (C), nursing home (N), hospital (H), WWTP influent, WWTP effluent (E), or surface water (SW)

Wastewater samples from C and N induced the greatest number of DEGs (Fig. 4), 1282 and 1427, respectively (− log_10_(*p*) > 4.0; false discovery rate, FDR < 0.01). In contrast, differential expression in samples H and I was much lower with 464 and 406 genes, respectively. Only two genes (*ncx-4* and F22B8.7) were differentially expressed in the nematodes treated with sample E and were both upregulated (1.1-fold for *ncx-4* and 1.5-fold for F22B8.7). Of these two genes, differential upregulation of F22B8.7 (1.4-fold) was also found in the sample I. Of the genes whose transcription levels (absolute-value expression) were changed more than fivefold (Fig. 5), most were found in nematodes exposed to C (166 DEGs) and N (101 DEGs) wastewaters, representing 13% and 7% of total DEGs of each sample, respectively. For samples H and I, 33 and 23 DEGs representing 8% and 7% of total DEGs of each sample were changed over fivefold. The two most upregulated genes for all wastewater were C23G10.11 (> 40-fold for samples C and N or > 20-fold for samples H and I) and B0222.4 (39-fold for C, 25-fold for H, 29-fold for N, and 23-fold for I). The decrease in expression level of T06C12.14 (40-fold for C and 15-fold for I) and Y49G5A.1 (19-fold for I and 17-fold for H) represented the most downregulated transcripts.

### Functional Analysis of Differentially Expressed Genes (DEGs)

**Fig. 6:**
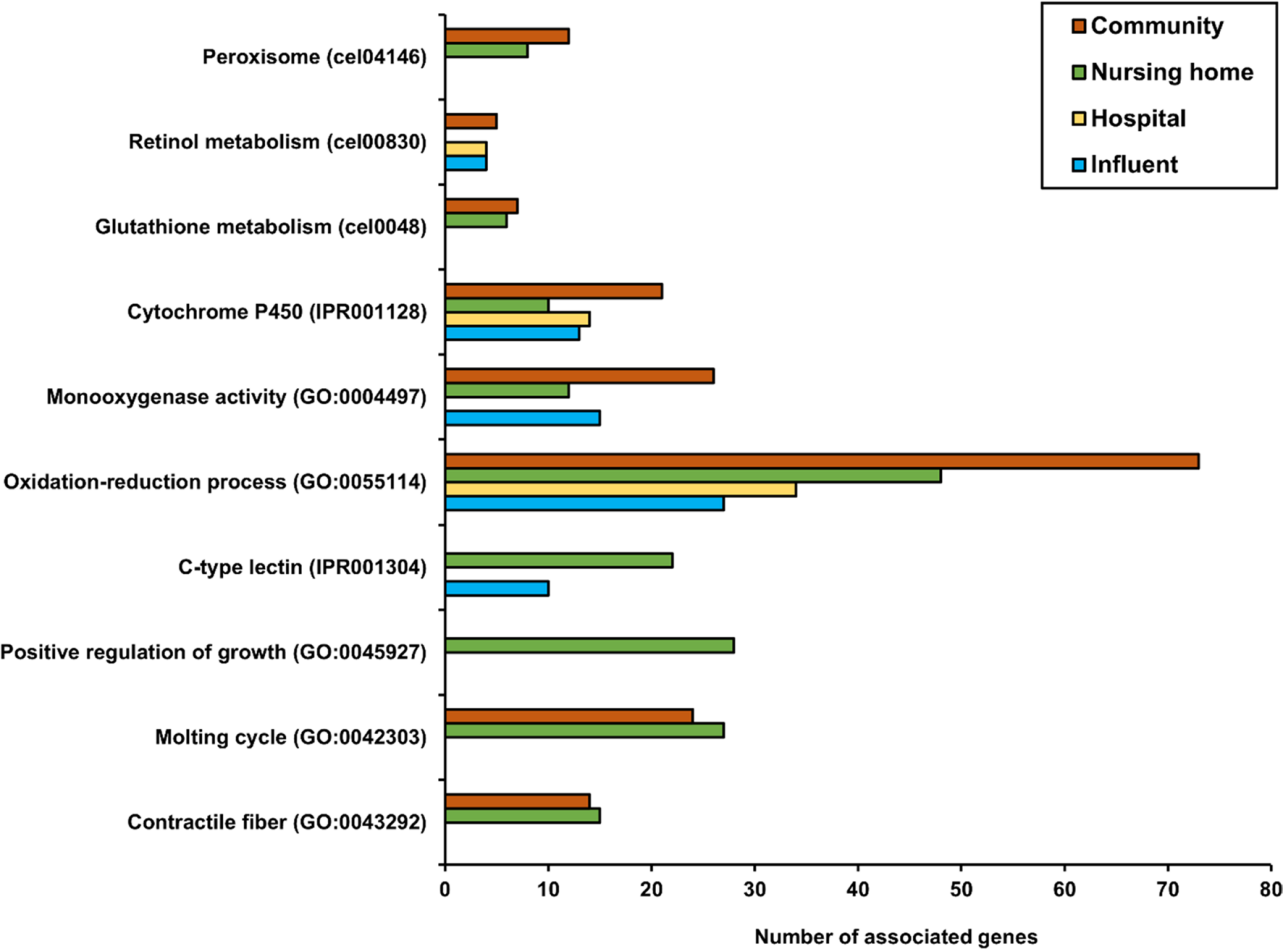
Some significantly upregulated genes for which enriched terms could be obtained [false discovery rate (FDR) < 0.05]. Full results of functional enrichment analysis are provided in Tables S3 and S4 of supplementary information

Gene ontology (GO) and domain enrichment analysis of DEG lists were carried out in DAVID software to identify the types of biological mechanisms underlying the nematode responses triggered by exposure to wastewater samples (Fig. 6 and Table S3). We identified a total of 36 genes encoding nuclear hormone receptors (NHRs) whose expression levels were affected by exposure. Of these genes, 10 transcripts (including *nhr-23* gene which is a critical regulator of the nematode growth and molting) were upregulated, while the other 26 genes were downregulated. Many upregulated genes were related to the nematode metabolic processes, especially those involved in the biotransformation (both phase I and phase II) of a wide range of substrates such as lipids, carbohydrates, and proteins. These biotransformation genes included those encoding cytochrome P450 (CYP), glutathione S-transferases (GSTs), UDP-glucuronosyltransferases (UGT), NADPH-cytochrome P450 reductase homolog (*emb-8*), and a number of genes annotated as FAD/NADP coenzymes. Cytochrome genes *cyp-25A1, cyp-25A2, cyp-29A2, cyp-33B1, cyp-35B1,* and *cyp-37A1* were upregulated in all wastewater samples. Transcriptional repression was found for pathways involved in the metabolism of purine and pyrimidine nucleotides and was identified in nematodes exposed to samples C and H. We also found DEGs involved in a peroxisomal pathway, including the transcripts of *acox-3*, *prx-3*, *prx-5*, *gstk-1*, *daf-22*, *ctl-2*, *ech-4*, *fard-1*, *acs-13*, C24A3.4, T20B3.1, and ZK550.6 genes upregulated by samples C and *prx-3*, C24A3.4, *daao-1*, *prx-14*, *ctl-2*, *ech-4*, *sod-1*, and *acs-13* upregulated by sample N. Genes annotated for oxidative stress response were found upregulated, including *pdi-2* and F09F3*.5* (in sample C), *pept-1* (in N), R08F11.7 (in C and N), and *col-61* (in C, H, and N samples).

Also genes involved in the *C. elegans* molting cycle processes were upregulated in C and N samples*.* These included the DEGs encoding collagen and cuticulin-based cuticle in the nematode. We also identified upregulation of many genes modulating growth processes in the nematodes treated with sample C. The *daf-*36 gene encoding a Rieske-like oxygenase, which is a component of *C. elegans* endocrine system, was upregulated in samples C, H, and N exposure, but not in sample I. The individual annotation (in DAVID software) of all DEGs, which responded to the wastewater samples, revealed several transcripts that can be linked to reproductive physiological processes in *C. elegans* (Table S4). Nevertheless, reproduction-related processes (GO:0000003) were not found among the significantly regulated processes as obtained by GO enrichment analysis. We also found in total 40 DEGs encoding C-type lectin (CLEC) proteins, which are related to the immune response in nematodes. Of these, 11 genes were differentially expressed in all wastewater samples including both upregulation (*clec-39, clec-52, clec-55, clec-57, clec-221,* and *clec-227)* and downregulation (*clec-45, clec-53, clec-62, clec-63, clec-147,* and *col-137)*.

### Validation of Microarray Data by RT-qPCR

**Fig. 7:**
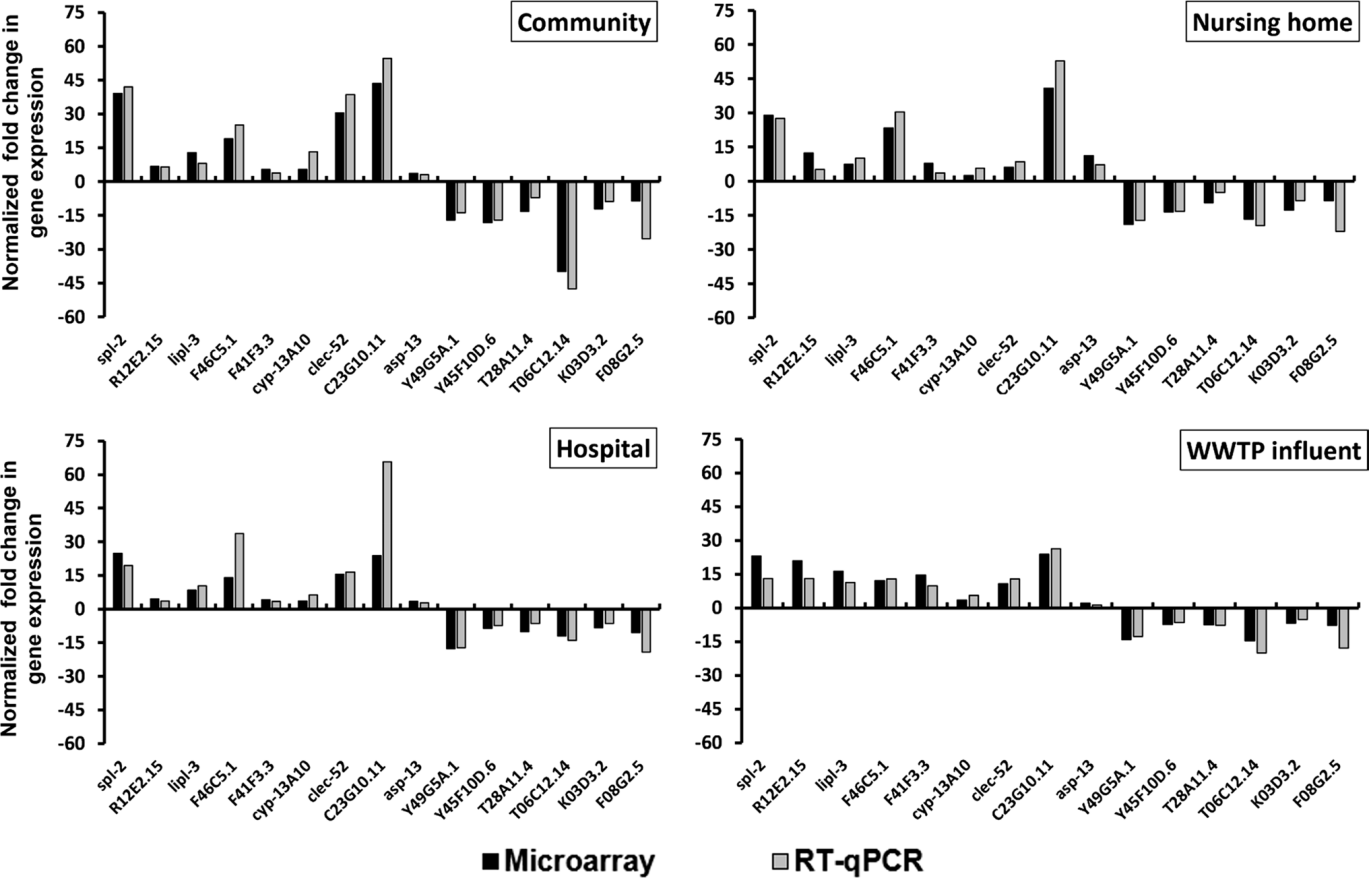
Validation of gene expression microarray results by reverse transcription polymerase chain reaction (RT-qPCR) for 15 target genes in two independent biological replicates using the RNA template from microarray samples. Negative values indicate downregulation and positive values upregulation of the target genes relative to two housekeeping genes (*tbg-1* and *par-5*) used to normalize the expression fold changes

To validate the microarray results, we conducted RT-qPCR for 15 target genes that were among the top most affected transcripts, among those regulated in all wastewater samples, or those specifically responding to one or two wastewater samples. Overall, RT-qPCR results correlated to the microarray results (Fig. 7).

## Discussion

In this study, we successfully applied a nematode-based assay using gene expression profiling in *Caenorhabditis elegans* to fingerprint wastewaters before and after treatment by a WWTP and effluent receiving surface waters. Several genes were differentially regulated following the exposure to wastewater samples, and this effect was absent in nematodes exposed to treated effluent as well as in effluent receiving surface water. The nematodes were exposed without extraction or preconcentration of water samples, except the removal of suspended solid materials by centrifugation. This means that bioanalysis with the water-exposed nematodes will especially indicate the total toxic potencies of bioactive pollutants (including hydrophilic compounds) that may be present in the tested samples, even at concentrations that could not yet be detected with chemical analysis.

Untreated and treated wastewater can typically contain a wide range of natural and synthetic chemical contaminants and reaction products and metabolites thereof (Cicek et al. 2007; König et al. 2017; Venkatesan and Halden 2014). The composition and type of contaminants present in each water source can vary depending on several factors (Khatri and Tyagi 2015). The most challenging substances to detect and quantify are hydrophilic compounds, which are hardly known and difficult to detect with existing chemical analytical techniques (Schwarzenbach et al. 2006). The exposure of nematodes to water samples containing hydrophilic compounds, which are invisible by chemical analyses, is expected to leave their signature in this invertebrate detectable by transcriptome analysis. In this study, gene expression profiling using microarray provides information about the total combined toxic potency specified per mechanism of action without the need to know the nature of the causative agents.

Although 209 genes were differentially regulated (77 upregulated DEGs and 132 downregulated DEGs) in all four types of wastewaters, these sample types also had specific DEGs that could be characteristic for the source. These included 31%, 4%, 35%, and 13% of the total DEGs specifically regulated in response to the sample C, H, N, and I exposure, respectively. There were also several DEGs regulated in the nematodes treated with the samples C, H, and N, but were not found in the sample I exposure. Compared with the total amount of DEGs found with each wastewater source, these genes comprised 74% for C-affected, 35% for H-affected, and 83% for N-affected DEGs (including the overlaps). The expression of these genes may be linked to substances that were diluted by the additional water from other sources (which accounted 97% of the total influent) such as stormwater runoff, seepage water, and water from other community households. It is also possible that the substances in wastewater sources were degraded or have reacted before reaching the influent. More detailed study, including more sampling (time) points and combining this with a tiered approach for screening and assessment of the contaminant mixtures, can reveal the most important bioactive compounds, their sources, and their fate. This is comparable to the approach of effect-directed analysis (EDA) utilizing the process similar to the toxicity identification evaluation (TIE) to identify unknown contributors to the mixture effects in water samples as described previously by Escher et al. (2021).

Only two genes were regulated in the nematodes treated with effluent, suggesting an efficient removal of bioactive pollutants by the WWTP, and none after emission of the effluent into the surface water. This means that the nematode assay could be developed into a bioanalytical tool for determining whether the toxic potency is below a threshold of ‘no indications for concern’. The small size of the nematodes and sensitivity of molecular endpoints potentially make the assay sensitive for ultra-low concentrations of contaminants. The aim, however, does not necessarily have to be to make the assay as sensitive as possible, but sensitive enough to be able to determine whether the possibly remaining contaminants do not pose a risk.

Another advantage of this small-scale bioanalytical in vivo tool is that the DEGs provide mechanism-based information on the combined toxic potency of the contaminants present, including the unknown hydrophilic compounds. In this study, genes related to metabolic processes were affected most. These included several genes involved in the metabolic pathways such as the *emb-8* gene encoding *C. elegans* NADPH-cytochrome P450 reductase homolog (EMB-8) which governs the nematode CYP-mediated metabolism (Kulas et al. 2008; Leung et al. 2010). There was also significant expression among the genes involved in the peroxisomal pathway, which is essential in the antioxidant defense system. Of these genes, *ctl-2* (Petriv and Rachubinski 2004), *sod-1* (Yanase et al. 2009), and *gsto-1* (Burmeister et al. 2008) are known for their central role in the detoxification of reactive oxygen species (ROS). Other genes annotated for oxidative stress response were upregulated, including *col-61, pdi-2, pept-1*, R08F11.7, and F09F3.5 transcripts. These observations do not imply a toxic risk per se, as explained by Leusch and Snyder (2015), but the involved genes do indicate exposure to compounds that trigger the organism’s defense mechanism.

Wastewaters have been shown to contain endocrine-disrupting compounds (Kusk et al. 2011), which are highly heterogeneous in source and nature (Pironti et al. 2021). Nematodes have been shown to be sensitive for the effects and mechanisms of endocrine-disrupting compounds as has been reviewed by Höss and Weltje (2007). The authors demonstrated evidence that many processes like molting or growth, regulated via hormonal pathways, are also operational in *C. elegans*. In our study, the differential gene expression profile of these pathways induced by wastewater, mostly in those originating from community and nursing home, indeed suggests the suitability of *C. elegans* to indicate endocrine active compounds. The DEGs included those required for molting, growth, and reproduction processes in the nematode, and especially well-known regulators of *C. elegans* development like *nhr-23* (Kouns et al. 2011), *unc-52* (Rogalski et al. 1995), and *daf-36* (Rottiers et al. 2006)*,* together with many of their downstream genes. This finding suggests the presence of endocrine disrupting substances in the tested wastewater samples and the absence thereof in the effluent and surface water samples. The application of bioassays in high-resolution effect-directed analysis has been recently demonstrated for the identification of endocrine-disrupting and mutagenic compounds in WWTP effluents and the river Meuse (Zwart et al. 2020).

Our study also identified differential expression of many genes contributing to the nematode innate immune system, especially those encoding C-type lectin (CLEC) proteins. This could be related to exposure of the nematodes to microorganisms from the wastewaters including pathogens that may trigger an immune response in the nematodes as previously reported by Irazoqui et al. (2010). Proteins encoded by the DEGs that we found in the wastewaters are associated with the innate immune mechanisms of invertebrates (Pees et al. 2016). The genes *clec-52, clec-70, clec-61, tag-38, acdh-1, myo-2,* F55G11.7, Y51H4A.5, and *unc-52*, also found in the outcome of our study, were linked to the *C. elegans* infection by the bacteria *P. aeruginosa* and *S. aureus* (Irazoqui et al. 2010). Among the 300 CLEC genes estimated to be present in the *C. elegans* genome (Takeuchi et al. 2008), our study showed that 40 CLEC genes responded to the wastewater exposure but not to effluent or surface water exposure. Noteworthy, *spl-2* that was among the top upregulated transcripts by all wastewaters is also involved in the nematode defense response to a gram-positive bacterium (Irazoqui et al. 2010). Further transcriptomic profiling of CLEC genes in *C. elegans* exposed to various pathogen types can provide gene markers that may specifically detect those pathogens in water sources.

## Conclusion

Overall, this study showed that gene expression profiling in *C. elegans* is a potential powerful tool for monitoring water-soluble pollutants in wastewaters. This bioanalytical assay especially is suitable for monitoring of the mechanism-specific toxic potency from bioactive pollutants (including hydrophilic compounds) since the nematodes can be directly exposed to even severely polluted wastewater samples without the need to pretreat or to dilute the samples. The results from this study showed a strong difference between polluted water and clean(ed) water samples in terms of gene expression profiles and intensity. Hence, our method can be used for monitoring the removal efficiency of (micro)pollutants during wastewater treatment and assessing the quality of the resulting effluent and receiving waters. In a tiered approach, this bioanalytical tool could help identify the most important bioactive compounds, their sources, and their fate. Also, the mechanistic profile of specific compounds of interest could be studied to possibly be able to identify, for instance, the presence of (recreational) drugs in wastewater. In addition, transcriptional profiles could be used to identify the presence of wastewater input or specific wastewater sources. It also is important to study the lowest induction level below which there is no indication for toxicological concern from hydrophilic compounds, compounds that are not yet easily detected, quantified, and assessed based on chemical analysis.

## Supplementary Information

Below is the link to the electronic supplementary material.

Supplementary file1 (XLSX 70 kb)

## Data Availability

The datasets generated during and/or analyzed during the current study are available in the ArrayExpress repository (E-MTAB-11260).
